# Learning protein representations with conformational dynamics

**DOI:** 10.1093/bioinformatics/btag254

**Published:** 2026-05-05

**Authors:** Dan Kalifa, Eric Horvitz, Kira Radinsky

**Affiliations:** Department of Computer Science, Technion—Israel Institute of Technology, Technion City, Haifa 3200003, Israel; Adaptive Systems and Interaction Group, Microsoft Research, Redmond, WA 98052, United States; Department of Biomedical Informatics and Medical Education, University of Washington, Seattle, WA 98195, United States; Department of Computer Science, Technion—Israel Institute of Technology, Technion City, Haifa 3200003, Israel

## Abstract

**Motivation:**

Proteins change shape as they work, and these changing states control whether binding sites are exposed, signals are relayed, and catalysis proceeds. Most protein language models (PLMs) pair a sequence with a single structural snapshot, which can miss state-dependent features central to interaction, localization, and enzyme activity. Studies also indicate that many proteins assume multiple, functionally relevant shapes, motivating approaches that learn from this variability.

**Results:**

We present DynamicsPLM, a PLM conditioned on ensembles of computationally generated conformations to derive state-aware representations. DynamicsPLM improves predictive performance across protein–protein interaction, subcellular localization, enzyme classification, and metal-ion binding. On a widely used protein–protein interaction benchmark, it achieves a four-point accuracy gain over the strongest baseline. On a curated test set enriched for proteins with multiple conformational states, the margin increases to eleven points. These findings argue for a shift from static to dynamics-aware modeling, in which conformational variability is treated as informative. By elevating conformational state to a central element of machine learning in protein biology, this work advances modeling toward mechanisms that better reflect how proteins operate in cells and provides a route to actionable hypotheses about when and how binding, signaling, and catalysis occur.

**Availability and implementation:**

Code, model weights, and inference scripts are available at https://github.com/kalifadan/DynamicsPLM (DOI: https://doi.org/10.5281/zenodo.17668302).

## 1 Introduction

Many proteins exist not as a single rigid fold but as a shifting ensemble of conformations, transitioning among distinct functional states as they bind substrates, undergo post-translational modifications, or interact with partner molecules (Henzler-Wildman and Kern 2007). These changes in structure—whether subtle domain flexing or large-scale allosteric transitions—are critical to the protein’s role in signaling, regulation, and catalysis. In recent work, generative models, trained on protein structures, molecular dynamics results, and experimental data, have been employed to make inferences about molecular dynamics, yielding protein equilibrium ensembles (Lu et al. 2025, Lewis et al. 2025, Sledzieski and Hanson 2026).

Despite advances in predicting different configurations and equilibria of conformations in protein interactions, protein language models (PLMs) that underpin much of today’s structure-based prediction rely on static structural snapshots. As an example, a great deal of research assumes a single AlphaFold-predicted coordinate set (Jumper et al. 2021) or Protein Data Bank (PDB) entry (Berman et al. 2000), overlooking that conformational heterogeneity can influence biological behavior (Lin et al. 2023, Su et al. 2024, Kalifa et al. 2025). This choice can introduce concrete inconsistencies in downstream features that are state-dependent, such as pocket accessibility, catalytic geometry, and interface exposure.

A motivating example is triosephosphate isomerase (UniProt P04789), whose active-site ω-loop (169–176) toggles between ‘open’ and ‘closed’ states. Open apo structures (e.g. PDB: 5TIM) admit substrate, whereas ligand-bound complexes adopt a closed loop that seals the pocket (e.g. PDB: 1NEY). The AlphaFold model (Jumper et al. 2021) for this protein predicts a closed loop with high confidence (pLDDT >85), despite the known open/closed ensemble of the protein. Conditioning models on this single prediction risks conflating ligand-induced closure with intrinsic properties of the protein and can bias predictions on multi-conformation targets.

To address this limitation, we introduce *DynamicsPLM*, a PLM that accounts for structural dynamics by integrating over multiple conformations via a dynamic embedding layer. The model is fully compatible with generative conformation models (e.g. BioEmu (Lewis et al. 2025)); their predicted conformations can be used directly as inputs to the dynamics embedding layer without other changes in the pipeline. We treat the conformation generator as independent and frozen: its conformations serve as inputs to the dynamics embedding layer, while we train only the embedding layer and the PLM encoders. This design avoids perturbing the conformation generator and prevents conflating gains from improved generation with gains from our fusion mechanism.

**Figure 1:**
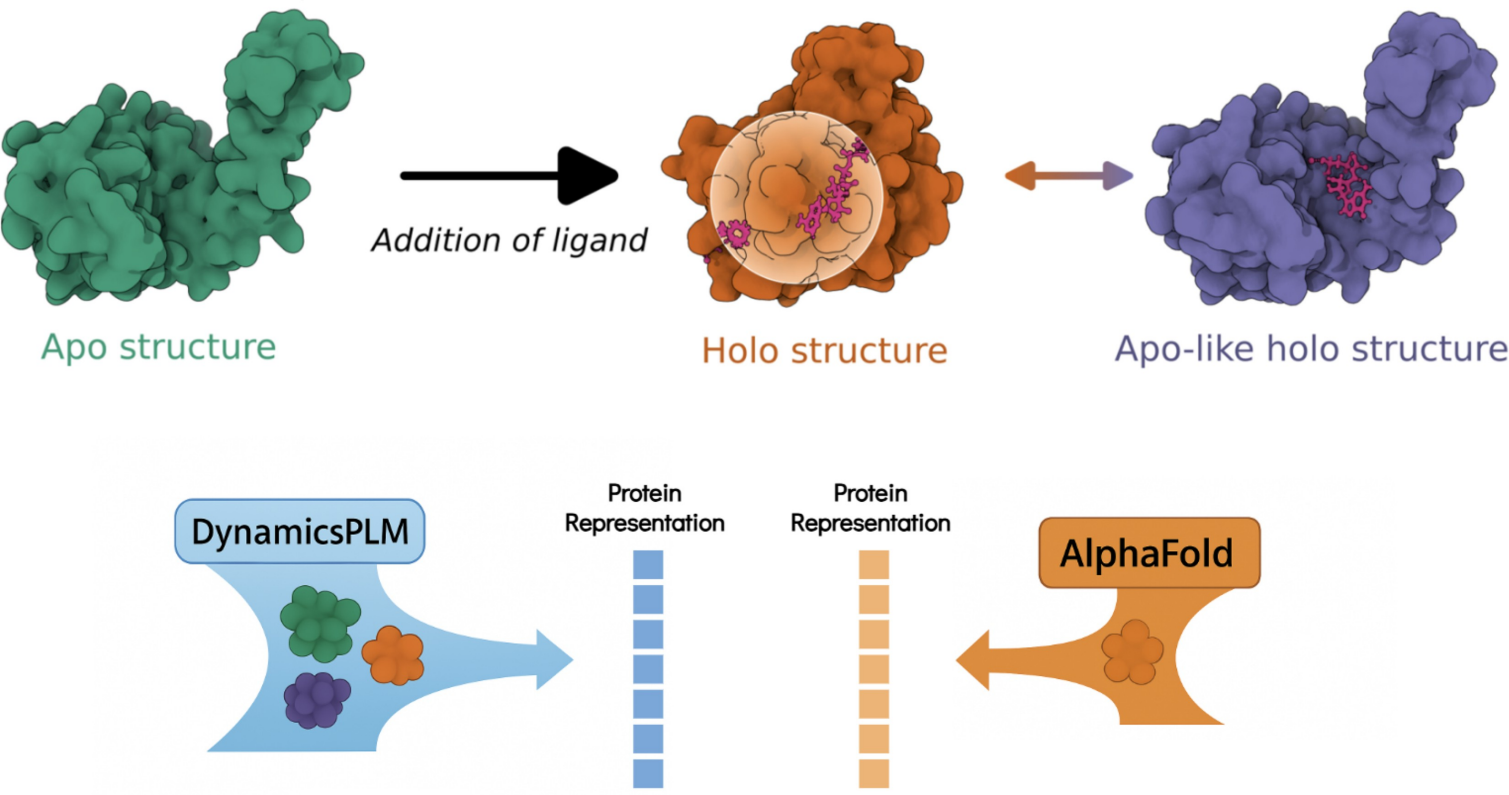
Conformational change enables interaction. Top: Conformation-dependent binding pocket of adenylate kinase (Ellaway et al. 2024): an *apo* conformation (left; PDB 6S36, green), ligand-bound *holo* conformation with a formed/expanded pocket (center; PDB 8CRG, orange), and an *apo-like holo* conformation that retains an apo-like global fold while accommodating the ligand (right; PDB 6F7U, purple). Bottom: Illustration of DynamicsPLM protein embedding that is built via conditioning on ensembles of conformations and learning state weights that emphasize binding-competent configurations while preserving alternatives. In contrast, single-structure pipelines (e.g. SaProt (Su et al. 2024)) rely on AlphaFold-derived models (Jumper et al. 2021) and use a single, static structure—often a holo-like conformation (≈70% of cases) (Saldaño et al. 2022), thus missing ensemble effects that are critical for interaction-dependent function.

As shown in Fig. 1, the methodology departs from prior PLMs in its core representation. Rather than conditioning on a single structure, DynamicsPLM constructs an ensemble of plausible protein conformations. Each conformation is discretized into structure tokens from a 3D alphabet produced by a VQ–VAE–pretrained autoencoder (van Kempen et al. 2024). These structure tokens are combined with amino acid tokens to form structure-informed sequences, which are then passed through our proposed dynamics embedding layer to yield per-residue, weighted, conformation-aware token embeddings.

We find that the resulting representation is ‘sensitive to both sequence context and conformational flexibility’, allowing the model to learn structure-conditioned embeddings that reflect the probabilistic nature of proteins. This design improves the model’s ability to resolve paralogs—proteins with near-identical sequences and the same overall fold but differing in the amplitude or occupancy of local motions (e.g. loop closures, hinge bends) and to identify functionally relevant regions defined not by static geometry but by their propensity for dynamical shifts.

Recent models such as AlphaFold 3 (Abramson et al. 2024) have substantially improved prediction of biomolecular complexes, including protein–protein and protein–ligand assemblies. However, AlphaFold 3 addresses a different objective from ours: given specified molecular inputs, it predicts candidate structures for the specified biomolecular assembly and ranks them using confidence estimates, rather than explicitly modeling ensembles of conformational states or their occupancies in solution. In many biological settings, the central challenge is not only recovering a plausible bound complex but representing the range of conformations a protein can access across functional states. DynamicsPLM is designed for this latter problem, learning reusable protein representations that encode conformational heterogeneity for downstream tasks such as interaction prediction, localization, and function prediction. Because AlphaFold 3 is applied to specified molecular inputs for each candidate complex, DynamicsPLM instead produces reusable protein representations that can be compared across many potential partners without repeated pairwise structure prediction.

A straightforward alternative is to encode several conformers separately and then average their embeddings. However, ‘such averaging collapses distinct structural modes into a single vector that corresponds to no single realized physical conformation’, muting signals tied to specific functional states (e.g. open versus closed loops, or ion-bound versus unbound pockets). In contrast, DynamicsPLM represents distributions of states rather than averages of states: for each residue, we construct a distribution over discrete structural microstates inferred from a conformational ensemble. This distribution approximates the local conformational free-energy landscape and preserves multi-modality, allowing the encoder to prioritize the state most relevant to the task at hand. We present a comparison (Section S4.2, available as supplementary data at *Bioinformatics* online) that contrasts our method with simple averaging of embeddings across generated conformers, which yields lower performance while incurring additional computational overhead.

We evaluate DynamicsPLM on several fundamental protein tasks: HumanPPI (human protein-protein interactions), metal ion binding, enzyme commission (EC), and subcellular localization prediction (DeepLoc). We ensure strict separation of close homologs by verifying that every protein shares at most ≤30% pairwise sequence identity with proteins in other splits. Across test sets, DynamicsPLM consistently outperforms the strongest baseline, improving accuracy by +4.0 points in HumanPPI, +1.9 in metal ion binding, and +1.6 in DeepLoc and increasing EC $F_{\max}$ by +0.9 points. All gains are statistically significant (*P* < .05, two-tailed paired *t*-test with Holm–Bonferroni correction). These gains are significant because the evaluated tasks underpin many real-world applications, including drug discovery, which relies on accurate modeling of protein-protein interactions, and the elucidation of disease mechanisms through subcellular localization prediction.

To assess sensitivity to conformational dynamics, we re-evaluate all methods on proteins with multiple experimentally determined conformations from the CoDNaS-Q database (Escobedo et al. 2022). In this protein dynamics subset, DynamicsPLM shows improvements over the strongest baselines: +11.1 points in HumanPPI (n=18), +3.85 in metal ion binding (n=101), +6.25 in DeepLoc (n=16), and +6.5 points in EC $F_{\max}$ (n=134). All gains are statistically significant (*P* < .05, two-tailed paired *t*-test).

These results suggest that conditioning on conformational ensembles may provide greater benefits for proteins experimentally confirmed to exhibit pronounced structural heterogeneity. However, the filtered subsets are relatively small, and therefore these results should be interpreted as indicative rather than definitive. In contrast, improvements are modest when conformational dynamics are limited. For proteins with only a single experimentally determined structure, performance remains comparable to strong baselines, suggesting that ensemble conditioning does not introduce noise in the absence of conformational diversity. Together, these results indicate that conditioning on conformational ensembles, rather than a single snapshot, yields more reliable, biologically grounded protein representations and should become a default strategy for structure-informed PLMs in applications ranging from interaction and localization prediction to drug discovery.

## 2 Materials and methods

### 2.1 Overview of DynamicsPLM

**Figure 2:**
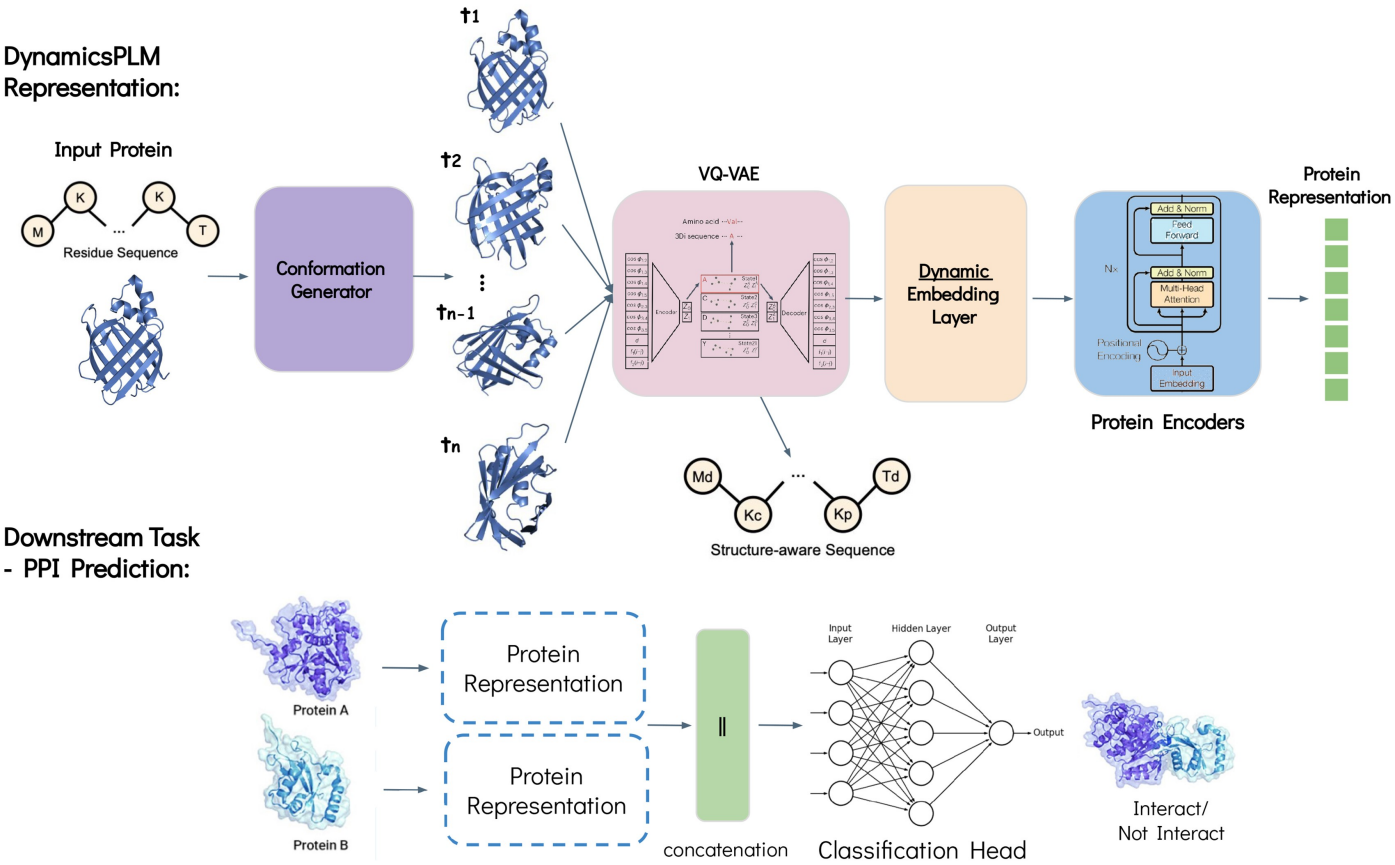
DynamicsPLM architecture. Top: The model is trained on amino acid sequences together with 3D structures predicted by AlphaFold2 (Jumper et al. 2021). We obtain multiple plausible conformations per protein via a conformation generator. For each conformation, we construct a structure-aware sequence by pairing residue tokens with discretized structure tokens produced by a VQ–VAE–pretrained autoencoder (van Kempen et al. 2024). The dynamics embedding layer then performs weighted, per-residue fusion across conformations estimated from token frequencies within the ensemble (normalized to sum to one), yielding a single sequence of conformation-aware token embeddings. The fused sequence is passed through the encoder layers to produce a refined protein representation. Bottom: Example of the downstream-task phase for the HumanPPI task, where the model predicts whether two given proteins interact.

DynamicsPLM is a broadly applicable PLM (see Fig. 2) that accounts for structural dynamics by integrating over multiple conformations via a unique dynamic embedding layer. The model is trained on amino acid sequences paired with 3D structures predicted by AlphaFold2 (Jumper et al. 2021). In a first stage, a generative conformation model proposes multiple plausible conformations per protein. We utilize RocketSHP (Sledzieski and Hanson 2026), a scale-efficient variant of BioEmu (Lewis et al. 2025) shown to reduce prediction error, though the framework remains compatible with alternative generators. For each conformation, we build a structure-aware sequence by pairing residue tokens with discretized structural tokens produced by a VQ–VAE autoencoder (van den Oord et al. 2017). The dynamic embedding layer then performs per-residue, cross-conformation fusion using ensemble-derived weights (normalized to sum to one), yielding a single sequence of conformation-aware token embeddings. The fused sequence is processed by transformer encoder layers initialized with pre-trained SaProt-650M weights (Su et al. 2024) to produce a refined protein representation, where SaProt is a large-scale general-purpose PLM trained on ∼40 million protein sequences and structures, and where protein structures are encoded into discrete tokens using Foldseek (van Kempen et al. 2024). Then, per downstream task, we incorporate task-specific classification heads to enable predictions for these tasks. See Section S1, available as supplementary data at *Bioinformatics* online for the full algorithmic details and implementation specifics.

### 2.2 Benchmark tasks

We assess the effectiveness of our approach for protein representation learning on a diverse set of tasks, selected according to the top-performing joint structure–sequence PLMs, structure-only PLMs, and sequence-only PLMs (Zhang et al. 2023a, 2023b, Su et al. 2024). These tasks span several biological fields, including protein-protein interaction prediction, localization prediction, and binding site classification (see Section S1.2 for detailed task descriptions and Section S2.1 for evaluation and dataset details, available as supplementary data at *Bioinformatics* online).

To ensure the validity of the results, we validate all downstream task splits by enforcing that sequences in one set share no >30% Needleman–Wunsch sequence identity with any sequence in the other sets. For the HumanPPI task, we use group-wise splits by protein, assigning all interactions of a given protein to a single split to prevent partner leakage; the 30% identity filter is applied at the protein-cluster level prior to pairing. This homology filtering ensures minimal overlap between training and evaluation data, minimizing the risk of data leakage.

Benchmark test sets are strictly held out. To minimize pretraining exposure from the conformation generator, we screened all test proteins against the RocketSHP (Sledzieski and Hanson 2026) pretraining corpus and removed any sequence with ≥30% identity to that corpus.

## 3 Results

We present DynamicsPLM’s empirical performance on a diverse set of benchmark tasks, under multiple conditions, demonstrating SOTA results (The code and datasets used for the statistical testing are freely available on this GitHub repository: https://github.com/kalifadan/DynamicsPLM). In addition to quantitative metrics, we provide curated experimental case studies that link performance gains with biochemical phenomena.

We conducted ablation studies across all benchmarks isolating key components of our model: (i) the weighting function (Section S4.1, available as supplementary data at *Bioinformatics* online), (ii) replacing the dynamic embedding layer with a mean-pooling operator (Section S4.2, available as supplementary data at *Bioinformatics* online), (iii) swapping the conformation generator for an alternative (Section S4.3, available as supplementary data at *Bioinformatics* online), and (iv) a validation that improvements arise from meaningful conformational diversity rather than noise by introducing a Gaussian perturbation control (Section S4.4, available as supplementary data at *Bioinformatics* online).

We report the mean and standard deviation of DynamicsPLM’s performance over five independent fine-tuning runs (with different random seeds) to ensure reproducibility (Table 1). Additionally, we validate the statistical significance of performance differences, using a two-tailed paired *t*-test with a 95% confidence level (*P* < .05), comparing observations from the tested models. Table S5, available as supplementary data at *Bioinformatics* online lists the raw and Holm–Bonferroni–adjusted *P*-values for DynamicsPLM across tasks. We report the maximum Holm–Bonferroni–adjusted *P* across these seven comparisons for the task.

**Table 1:** Experimental results on four downstream tasks.^a^^,^^b^^,^^c^

Model	HumanPPI	Metal Ion Binding	EC	DeepLoc (Subcellular)
	(Acc%)	(Acc%)	($F_{\max}$)	(Acc%)
MIF-ST	75.54	75.08	0.807	78.96
ESM-1b	82.22	73.57	0.864	80.33
ESM-2	76.67	71.56	0.868	82.09
ESM-3	83.60	72.70	0.871	76.30
GearNet	73.86	71.26	0.874	69.45
ESM-GearNet	84.09	74.11	0.882	82.30
SaProt	86.67	75.15	0.876	83.19
**DynamicsPLM**	**90.66** ${\;}^{*}\pm 0.55$	**77.01** ${\;}^{*}\pm 0.2$	**0.891** ${\;}^{*}\pm 0.005$	**84.79** ${\;}^{*}\pm 0.08$

a Statistically significant results (*P* < .05) using a two-tailed paired *t*-test across proteins in the test set (with Holm–Bonferroni correction across baseline comparisons for each task) are marked with an asterisk (*). For DynamicsPLM, we report the mean and standard deviation over five independent fine-tuning runs (different seeds; HumanPPI: n=180, Metal Ion Binding: n=665, EC: n=1604, DeepLoc: n=2747). The best result is highlighted in bold.

b Significance markers refer to Holm–Bonferroni-adjusted *P*-values. Full raw and adjusted *P*-values are reported in Table S5, available as supplementary data at *Bioinformatics* online.

c 95% confidence intervals for DynamicsPLM are provided in Table S4, available as supplementary data at *Bioinformatics* online.

### 3.1 Benchmark results

In Table 1, we compare the performance of DynamicsPLM with seven SOTA baseline methods (see Section S1.1, available as supplementary data at *Bioinformatics* online for details) across diverse biological downstream tasks. DynamicsPLM significantly outperforms all baselines, achieving the best scores on every task. We compute Cohen’s *d* effect sizes (Cohen 1969) to quantify the magnitude of these improvements and observe large effects (d>0.8) across the board. We attribute these gains to the inclusion of the dynamic modality.

DynamicsPLM delivers the largest gains where state dependence is central and maintains an edge on tasks with weaker or implicit dynamic signals, highlighting ensemble-aware representations as a robust and generalizable foundation for protein function prediction. HumanPPI shows the largest gains, presumably because protein–protein interactions are strongly state-dependent (Nada et al. 2024). Interfaces form or break as binding patches become exposed or buried, loops open or close, and allosteric shifts change local geometry. By conditioning on an ensemble of conformations rather than a single structure, DynamicsPLM captures these state-dependent features and their sequence determinants, yielding a four-point improvement compared to the strongest baseline (Su et al. 2024) in this task. In practice, this leads to better detection of partners mediated by flexible loops and termini, as well as by transient, ligand- or metal-stabilized interface motifs, effects that existing models often overlook.

We find that Metal Ion Binding benefits from ensemble conditioning at known coordination sites, that DeepLoc gains reflect subtle state-linked exposure patterns relevant to trafficking signals, and EC improves despite limited explicit state annotations—suggesting that the learned representations generalize even when dynamic cues are only indirectly present in downstream labels. We further examine the performance gaps and find that ensemble context increases prediction confidence without sacrificing calibration. Confidence stratification and reliability diagrams across tasks (Section S3.3, available as supplementary data at *Bioinformatics* online) show upward shifts in accuracy at matched confidence and improved calibration slopes. Overall, these results underscore the value of incorporating conformational ensembles into protein modeling.

We additionally include ESM3 (Hayes et al. 2025) as a contemporary large-scale multimodal baseline. ESM3 jointly models protein sequence, structure, and function using discrete tokens, but it does not explicitly represent conformational ensembles or residue-level state occupancies across multiple plausible conformations. In contrast, DynamicsPLM explicitly captures conformational heterogeneity and encodes residue-level distributions over structural microstates across conformations.

### 3.2 “Dynamic” proteins

To quantify the impact of conformational heterogeneity on downstream performance, we repeated the evaluation on a filtered subset of each task’s original test set (see Table 2). The subset includes only “dynamic” proteins, identified using CoDNaS-Q (Escobedo et al. 2022), a curated dataset of proteins with multiple experimentally determined structures (i.e. conformers) that quantifies backbone variability across the ensemble. For each protein, CoDNaS-Q provides pairwise backbone deviations (using Cα RMSD differences), enabling us to select cases with non-trivial conformational diversity. Exact filtering criteria and per-task subsets are detailed in Section S2.2, available as supplementary data at *Bioinformatics* online.

**Table 2:** Experimental results for SaProt and DynamicsPLM on four downstream tasks, evaluated on a filtered subset of each task’s original test set.^a^^,^^b^

Model	HumanPPI	Metal Ion Binding	EC	DeepLoc (Subcellular)
	(Acc%)	(Acc%)	($F_{\max}$)	(Acc%)
SaProt	88.89	76.92	0.858	68.75
**DynamicsPLM**	100.0^*^	80.77^*^	0.923^*^	75.00^*^

a The subset includes only “dynamic” proteins^c^^,d^, identified by their known conformational ensembles and pairwise Cα RMSD differences based on the CoDNaS-Q dataset (Escobedo et al. 2022).

b Statistically significant results (*P* < .05) using a two-tailed paired *t*-test across proteins in the test set are marked with an asterisk (*). Sample sizes after filtering: HumanPPI (n=18), Metal Ion Binding (n=101), EC (n=134), DeepLoc (n=16). The best result is highlighted in bold.

c Due to the small subset sizes, we additionally applied exact McNemar tests and bootstrap confidence intervals. Improvements on HumanPPI and DeepLoc remained consistent under these small-sample analyses, supporting the observed results.

d Exact filtering criteria and per-task subsets are detailed in Table S3, available as supplementary data at *Bioinformatics* online, with further explanations on the dataset creation.

DynamicsPLM outperforms SaProt (Su et al. 2024), the strongest baseline, across all benchmarks. The results on the dynamic subset follow the same trends observed in the full test set, with larger relative gains on proteins exhibiting conformational heterogeneity. However, as the dynamic subset is small, these results should be interpreted as a targeted analysis rather than a definitive benchmark. Nevertheless, the observed trend is consistent with the hypothesis that state-dependent biology benefits from ensemble-based representations.

The largest improvement appears on HumanPPI, consistent with the main benchmark, with a gain of +11.11 points (100.0% versus 88.89%), for n=18. Other tasks benefit as well, with improvements of +3.85 points on Metal Ion Binding for n=101, +6.5 on EC for n=134, and +6.25 points on DeepLoc for n=16; all differences are significant (p<0.05).

The dynamic-subset analysis isolates the value of ensemble conditioning: when proteins are known to sample multiple states, predictions improve markedly across tasks. Practically, these findings support three recommendations. First, when multi-conformer data (experimental or molecular dynamics derived) are available, ensemble-aware encoders should be preferred. Second, benchmarks and evaluation protocols should preserve conformational diversity rather than collapsing to a single representative structure. Third, simple triage rules (e.g. RMSD thresholds or bound/unbound flags) can identify cases where ensembles are most likely to yield benefits.

### 3.3 Curated experimental case studies

To underscore the design and practical relevance of DynamicsPLM, we examine model outputs on a curated subset of human protein pairs with prior *in vivo* or *in vitro* experimental evidence for protein–protein interactions (Mishra et al. 2006). We present two case studies that highlight complementary error modes addressed by ensemble conditioning: (i) recovering a state-dependent true interaction and (ii) suppressing a biologically implausible pair. In each case, we compare DynamicsPLM with the top-performing single-structure PLM baseline (Su et al. 2024) to show how conformational ensembles reveal binding-competent interfaces that a single conformer may miss. We also evaluated a recently reported (2025) interaction (Schrecker et al. 2025) to test whether DynamicsPLM captures state-dependent interfaces and can predict interactions beyond our historical datasets.

We additionally evaluate AlphaFold 3 (Abramson et al. 2024) as a structure-based interaction baseline. Since AF3’s confidence scores are well calibrated with structural accuracy (Abramson et al. 2024), we use ipTM as a binary interaction predictor (Bryant et al. 2022, Evans et al. 2022), calling a pair interacting if ipTM ≥0.6 and non-interacting otherwise.

#### 3.3.1 Interacting pair: ATG10—ATG7

ATG10 (UniProt: Q9H0Y0) is an E2-like conjugating enzyme, and ATG7 (UniProt: O95352) is an E1-like activating enzyme in the ATG12→ATG5 autophagy pathway. Structural studies indicate that ATG7–ATG10 recognition is state-dependent, with noncanonical E1–E2 contacts and loop repositioning between unbound and bound forms (Kaiser et al. 2012, 2013). The ground truth label for these proteins is a confirmed interaction. DynamicsPLM predicts interaction with probability (0.7903), whereas the single-conformer baseline (i.e. SaProt (Su et al. 2024)) predicts non-interaction (wrong label) with a high probability of (0.600). We infer that evaluating multiple plausible conformations allows the model to capture binding-competent arrangements—surfaces on ATG7 and ATG10 that become complementary only in certain states, which may be under-represented by any single structure. We note that AlphaFold 3 (Abramson et al. 2024) also produced a plausible complex for the specified ATG7–ATG10 pair, but with only moderate confidence in the interface (ipTM =0.62, pTM =0.55). This illustrates that, even when a plausible bound-state model can be generated, each AlphaFold prediction represents a single structural hypothesis, which may not fully capture the range of conformations relevant to this interaction.

#### 3.3.2 Non-interacting pair: MDM4—GCSAM

MDM4 (UniProt: O15151) is a nuclear regulator of p53 with a peptide-binding N-terminal pocket and a C-terminal RING domain that heterodimerizes with MDM2; multiple partner-specific states have been described (Marine and Jochemsen 2016). GCSAM, also known as HGAL (UniProt: Q8N6F7), is a germinal-center B-cell adaptor localizing to the plasma membrane and cytoplasm, with ITAM/SH2-binding motifs that recruit kinases and modulate B-cell receptor signaling (Jiang and Lossos 2023). Here, the ground truth is non-interaction. DynamicsPLM predicts non-interaction with probability (0.7469), while the single-conformer baseline (i.e. SaProt (Su et al. 2024)) predicts interaction (wrong label) with high probability (0.8972). We infer that scanning across conformers did not reveal a solvent-exposed, conformer-consistent interface on MDM4 compatible with a membrane adaptor such as GCSAM, and the ensemble model accordingly lowers the score. AlphaFold 3 yields ipTM =0.25 and pTM =0.31 for this pair, corroborating the non-interaction prediction across modeling approaches.

#### 3.3.3 Interacting pair: TMEM9—CLCN3 (recent, membrane-state dependent)

A recent study (2025) reports that TMEM9 (UniProt: Q9P0T7) directly binds and inhibits the endosomal chloride-proton exchanger CLCN3 (UniProt: P51790) (Schrecker et al. 2025). Cryo-EM resolved the TMEM9–CLCN3 complex and showed that PI(3,5)P_2_ stabilizes the interface, indicating a membrane lipid–dependent, conformation-specific association. Cell-based assays in human cells corroborate the complex and its regulatory effect. On this pair, DynamicsPLM assigns a high interaction probability (0.7205), whereas a single-structure baseline predicts non-interaction (0.5963). We interpret this discrepancy as DynamicsPLM capturing multiple conformations—including lipid-stabilized, binding-competent states—that a single static structure under-represents, which is crucial for understanding this interaction.

AlphaFold 3 yields ipTM =0.26 and pTM =0.29 for this pair, both well below the accepted failure threshold (Bryant et al. 2022), and predicts ‘non-interaction’, an incorrect call given the experimentally confirmed complex (Schrecker et al. 2025). This is consistent with the known biology: the TMEM9–CLCN3 interface is lipid-stabilized and conformation-specific, a setting where static co-folding without ensemble sampling may not recover the binding-competent state and where ensemble-aware representations may offer complementary signals.

#### 3.3.4 Experimental conclusions

DynamicsPLM provides informative variation: it elevates positives that appear only in specific states and suppresses negatives that lack a viable interface in any state. By conditioning on conformational ensembles, DynamicsPLM elevates state-dependent true interactions and suppresses pairs lacking a viable interface across states, improving performance. Because our cases are grounded in curated *in vivo* or *in vitro* evidence, the model’s high-confidence outputs translate into concrete, testable hypotheses. In practice, this helps prioritize experiments and reduce cross-compartment false positives, linking representation learning to bench-ready validation plans.

## 4 Discussion

We introduced DynamicsPLM, a structure-informed PLM that integrates information across conformational ensembles. Treating state as part of the representation improves prediction across interaction, localization, and enzyme-function tasks, with the largest gains where biology is demonstrably state-dependent (e.g. protein–protein interfaces). As a core contribution, we introduce residue-wise ensemble conditioning that preserves multi-modal conformational occupancy via token histograms and learns to select function-relevant modes, outperforming encode-then-average baselines. In our evaluations, these gains are accompanied by more reliable confidence estimates, indicating improvements beyond simple threshold effects.

A practical attribute of DynamicsPLM is modularity. The protein conformation source can operate independently and be revised or replaced without architectural changes to the overall representation, enabling rapid adoption of improved generators.

The analysis of boosts in the conformational dynamics subset clarifies when consideration of ensembles matters most: the gains in predictive power are seen precisely on proteins where there is independent evidence for multiple conformational states—where biology depends on proteins assuming distinct changes in structure. This suggests concrete guidance: it is valuable to harness ensemble-aware encoders when bound–unbound pairs or simple geometric criteria indicate conformational diversity and to avoid collapsing benchmarks to a single representative structure.

Although this work focuses on protein-centric tasks, DynamicsPLM could be extended to protein–ligand modeling scenarios. In its current form, the model does not explicitly embed ligands but instead captures protein conformational heterogeneity, which may reflect ligand-dependent states when these are present in the conformational ensemble. This makes protein–ligand tasks such as binding affinity prediction, pocket identification, and ligand-specific interaction modeling promising directions for future work.

### 4.1 Limitations

We note several limitations in the methodology and experiments. First, ground-truth state labels are scarce: for many proteins, it is unknown which conformations are biologically active under the conditions implied by the downstream labels. Consequently, the approach depends on an external conformer generator whose coverage and biases may vary across families. Second, the backbone RMSD used for screening is an imprecise surrogate for functional state—global motions can inflate RMSD without changing activity, while local rearrangements that gate binding may occur with little change in RMSD. Third, the dynamic subsets are relatively small for some tasks, reflecting limited availability of experimentally determined conformers. In particular, the HumanPPI and DeepLoc subsets contain a limited number of dynamic proteins, which reduces statistical power and increases susceptibility to Type II error. Therefore, improvements observed on these subsets should be interpreted as indicative rather than definitive. In contrast, the Metal Ion Binding and EC subsets are substantially larger and provide more reliable estimates. Future work should expand dynamic benchmarks with curated experimental ensembles and larger collections of experimentally observed conformations.

## Supplementary Material

btag254_Supplementary_Data

## Data Availability

Code, datasets, model weights, and inference scripts are available at https://github.com/kalifadan/DynamicsPLM (DOI: https://doi.org/10.5281/zenodo.17668302).
