# Supplementary material for: Learning protein representations with conformational dynamics
Source: Bioinformatics. 2026 May 5;42(5):btag254. doi: 10.1093/bioinformatics/btag254 (PMC13197115; doi:10.1093/bioinformatics/btag254)
Supplement: btag254_Supplementary_Data [file btag254_supplementary_data.pdf]

S1 DynamicsPLM algorithm

We introduce DynamicsPLM, a broadly applicable PLM that accounts for structural dynamics by integrating over multiple conformations via a unique *dynamic embedding layer*. The full implementation details, including computational complexity analysis, are presented in Supplementary Section S1.3.

S1.0.1 Structure-histogram vector

Let a protein $p = (S, R)$ be defined by its amino acid sequence $S = (s_1, s_2, \ldots, s_L)$ and its 3D structure R, where $s_i \in \mathcal{V}$ denotes the i-th residue and $\mathcal{V}$ is the standard residue alphabet. Given the sequence and structure, a conformation generator produces K possible conformers $\{R^{(k)}\}_{k=1}^{K}$.

Each conformer R is discretized into structure tokens of a 3D alphabet $\mathcal{F}$ of size M, produced by a VQ–VAE–pretrained autoencoder (van Kempen *et al.*, 2022), yielding per-residue structure tokens $(f_1^{(k)}, \ldots, f_L^{(k)}) \in \mathcal{F}^L$.

Then, we compute for every position i, a structure-histogram vector:

$$\mathbf{w}_i \in \mathbb{R}^M, \quad w_i[a] = \frac{1}{K} \sum_{k=1}^{K} \mathbf{1}\{\, f_i^{(k)} = a \,\}$$

Where $w_i[a] \geq 0 \;\; \forall a \in \mathcal{F}$ and $\sum_{a \in \mathcal{F}} w_i[a] = 1$.

This vector represents the empirical distribution over structure tokens at i across the ensemble. We refer to $\mathbf{W} = (\mathbf{w}_1, \ldots, \mathbf{w}_L)$ as the SHP tensor (structure-histogram probabilities).

S1.0.2 Dynamic embedding layer

We maintain a learnable 3D embedding table $\mathbf{E} \in \mathbb{R}^{|\mathcal{V}| \times M \times D}$ that contains a vector for every (amino acid, structure token) pair. We leverage the pre-trained SaProt-650M (Su *et al.*, 2024) embeddings to initialize this table, leveraging its strong contextual understanding of structure-aware sequences.

For residue $s_i \in \mathcal{V}$ we form a structure-weighted embedding:

$$\mathbf{e}_i^{\text{dynamic}} \;=\; \sum_{a \in \mathcal{F}} \mathbf{w}_i[a] \cdot \mathbf{E}[s_i, a] \;\in\; \mathbb{R}^D$$

Let $\mathbf{e}_i^{\text{seq}}$ be the pre-trained embedding associated with the input token at i (i.e., an amino acid token). We fuse the two embeddings with a fixed convex combination to produce the final embedding, combining the sequential embedding with the dynamic one:

$$\mathbf{e}_i \;=\; \lambda \cdot \mathbf{e}_i^{\text{dynamic}} \;+\; (1-\lambda) \cdot \mathbf{e}_i^{\text{seq}}$$

This aggregation yields per-residue, weighted, conformation-aware protein embedding $\mathbf{e}_p = (e_1, \ldots, e_L) \in \mathbb{R}^{L \times D}$.

S1.0.3 End-to-end pipeline

During training, each protein is passed through the dynamic embedding layer, producing a dynamic protein embedding e_p. Then, multiple encoder layers produce a contextual protein representation $z_p \in \mathbb{R}^{L \times D}$, where L is the sequence length and D is the feature dimension. After the encoder layers, task-specific classification heads are incorporated to enable predictions for the specific downstream tasks.

We initialize the encoders with a large pre-trained PLM, SaProt-650M (Su *et al.*, 2024), leveraging its strong contextual understanding. Using SaProt allows us to fairly isolate and assess the contribution of dynamics conformation integration. Alternatively, other advanced PLMs can also be used within the proposed framework.

The model is fully compatible with other generative conformation models (e.g., BioEmu (Lewis *et al.*, 2025)); their predicted conformers can be used directly as inputs to the dynamics embedding layer without any changes to the pipeline. We present such a replacement in the Supplementary Section S4.3.

We treat the conformation generator as frozen: its conformations serve as inputs to the dynamics embedding layer, while we train only the dynamic embedding layer and the PLM encoders. This design avoids perturbing the conformation generator and prevents conflating gains from improved generation with gains from our fusion mechanism.

To strengthen our key design choices, we present ablation tests examining the weighting function (Supplementary Section S4.1), replacing the dynamic embedding layer with a mean-pooling operator (Supplementary Section S4.2), and replacing the generative conformation model with an additional generator (Supplementary Section S4.3).

S1.1 Baselines

Incorporating SOTA models as baselines and following SaProt (Su *et al.*, 2024) for fair comparison, we include ESM-1b (Rives *et al.*, 2019), ESM-2 (Lin *et al.*, 2023), and ESM-3 (Hayes *et al.*, 2025), which are considered top-performing sequence-based models. Structure-based baselines include GearNet (Zhang *et al.*, 2023a) and MIF-ST (Yang *et al.*, 2022), while ESM-GearNet (Zhang *et al.*, 2023b) serves as a representative joint sequence–structure model. We also evaluate against SaProt itself (Su *et al.*, 2024), the current leading PLM that incorporates AlphaFold-derived structure tokens.

S1.2 Tasks

We provide a comprehensive overview of the downstream tasks, selected according to the top-performing joint structure–sequence PLMs, structure-only PLMs, and sequence-only PLMs (Su *et al.*, 2024; Zhang *et al.*, 2023b,a), to rigorously evaluate the effectiveness of our proposed approach for protein representation learning.

S1.2.1 Protein-protein interaction prediction

Reliable detection of protein–protein interactions (PPIs) is essential for deciphering cellular processes and uncovering therapeutic targets, especially when the interactions have not been previously characterized (Humphreys *et al.*, 2021). We use the HumanPPI dataset from the PEER benchmark (Xu *et al.*, 2022) to evaluate binary interaction prediction between protein pairs. Following established best practices in this task (Xu *et al.*, 2022; Su *et al.*, 2024), we report accuracy as the primary evaluation metric.

S1.2.2 Protein function prediction

We evaluated protein function using the Metal Ion Binding (Hu *et al.*, 2022) task, a binary classification task designed to predict the presence of metal ion binding sites within a protein, evaluated by accuracy, which is the common practice in this task (Dallago *et al.*, 2021; Su *et al.*, 2024) for practical applications.

Table S1. Benchmark dataset statistics for the benchmark tasks.

**Dataset**	**Category**	**Metric**	**Train**	**Valid**	**Test**
HumanPPI (Xu *et al.*, 2022)	PPI Prediction	Accuracy	26319	234	180
Metal Ion Binding (Hu *et al.*, 2022)	Protein Function Prediction	Accuracy	5067	662	665
EC (Gligorijević *et al.*, 2021)	Protein Annotation Prediction	$F_{\max}$	13089	1465	1604
DeepLoc (Subcellular) (Armenteros, 2017)	Protein Localization Prediction	Accuracy	8747	2191	2747

S1.2.3 Protein localization prediction

We adopt the DeepLoc dataset (Armenteros, 2017), a subcellular localization task, including a 10-class multiclass classification task. We use accuracy as the primary performance metric following similar studies in this field (Su *et al.*, 2024).

S1.2.4 Protein annotation prediction

We evaluate protein functional annotation using the Enzyme Commission (EC) number prediction task from the DeepFRI benchmark (Gligorijević *et al.*, 2021). This task is formulated as a multi-label classification problem, where each protein may be assigned one or more EC labels. $F_{\max}$ score is used for evaluation.

S1.3 Implementation details

We utilized the pre-trained SaProt-650M (Su *et al.*, 2024) as the base protein encoder, and its pre-training embedding to initialize our learned embedding table. The hyperparameters of DynamicsPLM (summarized in Supplementary Table S2) were selected via grid search based on performance on the validation set of the training dataset. As a result, the weight of the dynamic component in the embedding layer is set to $\lambda = 0.5$. Also, the number of conformations generated per protein is set to $K = 20$. The dimensionalities of the protein representations are set to $D = 1280$. To accommodate long protein sequences, inputs are truncated to a maximum of 1,024 tokens. All training is performed using mixed-precision arithmetic to improve memory efficiency and computational throughput.

In addition, we incorporate task-specific classification heads to enable predictions of downstream tasks, following SaProt (Su *et al.*, 2024) settings, to ensure fair comparisons.

Table S2. Training hyperparameters for DynamicsPLM.

**Parameter**	**Value**
Optimizer	AdamW
β_1, β_2	0.9, 0.98
Weight decay	0.01
Learning rate	2×10^{-5}
LR schedule	Fixed
Warmup steps	–
Decay start / end	–
Training epochs	Task-specific
Batch size	64

S1.3.1 Computational complexity

All experiments were conducted on 4×NVIDIA A100 (80 GB) GPUs, with all run-time variables specified in our publicly available code. DynamicsPLM fine-tuning required approximately 24 hours (∼96 GPU-hours) per downstream task. The primary overhead scales with K, the number of conformations generated per protein, and with the depth of the encoder stack. In contrast, SaProt (Su *et al.*, 2024) was pre-trained from scratch for roughly three months on 64 GPUs. By reusing SaProt's pretrained embeddings as initialization and integrating dynamic context via a lightweight dynamic embedding layer, DynamicsPLM achieves an approximate 99% reduction in training cost relative to full PLM pre-training.

At inference time, DynamicsPLM introduces modest overhead. SaProt encodes at ≈ 0.014 seconds per 1,000 residues, whereas DynamicsPLM requires ≈ 0.016 seconds for the same input, an increase of about 12.5%. In addition, conformational ensemble generation using RocketSHP introduces a one-time pre-processing cost of ≈ 0.06 seconds per 1,000 residues. Thus, the total end-to-end inference time for a novel protein is ≈ 0.076 seconds per 1,000 residues. To avoid redundant computation, we generate conformations once per protein and cache them for reuse.

S2 Datasets overview

S2.1 Benchmark datasets

Supplementary Table S1 summarizes the benchmark datasets, including task categories, evaluation metrics, and the sizes of training, validation, and test splits.

S2.2 Dynamic proteins datasets

To isolate test cases where conformational state is most likely to affect function, we intersect each benchmark's test proteins with CoDNaS-Q (Escobedo *et al.*, 2022)—a curated resource that aggregates multiple experimentally determined conformers per protein and reports conformational diversity via the maximum pairwise C_α-RMSD across conformers. We then retain proteins satisfying two criteria: (i) a minimum number of conformers and (ii) a non-trivial range of pairwise C_α-RMSD (thresholds below). Requiring ≥ 3 conformers (or ≥ 2 for DeepLoc, where coverage is sparser) stabilizes diversity estimates and follows prior practice of quantifying state variability across multiple structures rather than single pairs.

Our thresholds are informed by structural biology conventions and prior analyses (Escobedo *et al.*, 2023; Monzon *et al.*, 2017), combined with distributional statistics on our test proteins to exclude outliers. Very small backbone deviations are typically attributable to thermal/experimental fluctuation and are not interpreted as distinct states. Accordingly, we set a lower bound of 0.4 Å on the C_α-RMSD range to exclude trivial noise while admitting subtle but meaningful changes.

Results are robust to small perturbations: varying RMSD thresholds by ±0.1 Å and the conformer-count criterion by ±1 changes subset sizes but leaves all qualitative conclusions unchanged (same direction of effects and significance). Importantly, we preserve the original dataset labels and splits;

Table S3. Dynamic-subset filtering per task. "CoDNaS overlap" counts test-set proteins present in CoDNaS-Q (Escobedo *et al.*, 2022) before filtering. RMSD refers to the range of pairwise C_α-RMSD across conformers.

Task	Original test	CoDNaS overlap	Filtering criteria	Final subset
HumanPPI	180	18	RMSD $\geq$ 0.4 Å; #conformers $\geq$ 3	18
Metal Ion Binding	665	138	RMSD $\geq$ 0.4 Å; #conformers $\geq$ 3	101
EC	1604	230	RMSD $\geq$ 0.4 Å; #conformers $\geq$ 3	134
DeepLoc (subcellular)	2747	27	RMSD $\geq$ 0.4 Å; #conformers $\geq$ 2	16

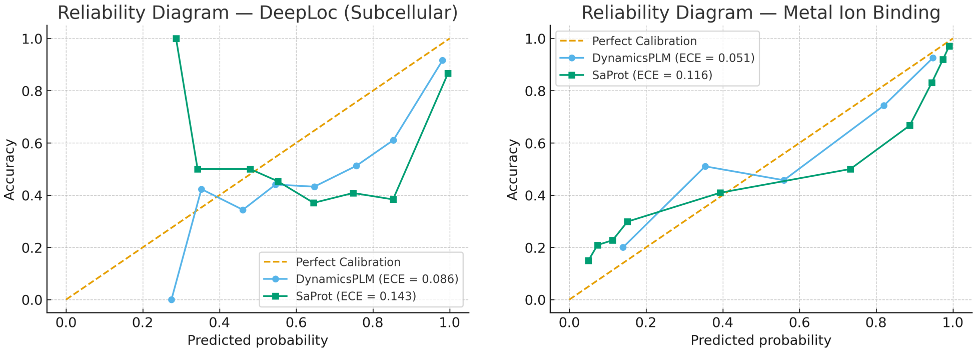

(a) DeepLoc (Subcellular)

(b) Metal Ion Binding

Fig. S1. Reliability diagrams for DeepLoc (Subcellular) and Metal Ion Binding. DynamicsPLM (blue) demonstrates better calibration than SaProt (green) in both tasks. In these classification tasks, DynamicsPLM achieves lower ECE while closely following the perfect line.

we only select test proteins that meet the criteria. Summary statistics are shown in Table S3.

S2.2.1 Sensitivity to Fine-Grained Conformational Differences

To further assess the resolution limits of the SHP representation, we analyzed proteins from the dynamic CoDNaS-Q subset (Table S3) containing multiple experimentally determined conformers. This analysis specifically targets fine-grained structural variability, where conformational differences are small and therefore challenging for discretized structural representations.

We selected proteins exhibiting conformational variability in the range of 0.4–0.7 Å Cα-RMSD, corresponding to near-sub-angstrom structural differences. This subset included 78 proteins with a total of 214 experimentally determined conformers, yielding an average of 2.7 conformers per protein. These conformers represent closely related structural states with minimal backbone deviations, providing a stringent setting for evaluating the sensitivity of SHP embeddings.

For each protein with K conformers, we calculated an SHP embedding that captures the residue-level distribution of structural tokens between conformations. We quantified variability using the mean per-residue variance of SHP weights across conformers. Despite the small structural deviations between conformers, we observed consistent, non-trivial SHP variance across residues, indicating that the SHP representation preserves fine-grained conformational variability rather than collapsing highly similar structural states.

These results demonstrate that SHP embeddings retain sensitivity to near-sub-angstrom conformational differences and help define the practical resolution limits of DynamicsPLM on experimentally observed dynamic proteins.

S3 Statistical and reliability evaluation

S3.1 Statistical significance tests

We perform two-tailed paired t-tests across proteins in the test set to assess whether DynamicsPLM significantly outperforms each baseline for a given task. For each task, raw p-values (DynamicsPLM vs. each of seven baselines) are adjusted using the Holm–Bonferroni procedure over that task's baseline comparisons. In Supplementary Table S5 we list the raw p-values and report the maximum Holm–Bonferroni–adjusted p across those comparisons. Statistically significant differences ($p < 0.05$) indicate that the observed performance improvements are unlikely to have occurred due to stochastic variation.

S3.2 Confidence intervals

To provide a clearer estimate of uncertainty across the five independent fine-tuning runs, we report 95% confidence intervals (CIs) for DynamicsPLM alongside the mean and standard deviation (Supplementary Table S4). Confidence intervals are computed using a two-tailed t-distribution with $n = 5$ (independent fine-tuning runs with different seeds).

Table S4. Mean ± standard deviation and 95% confidence intervals for DynamicsPLM across the benchmark tasks (five fine-tuning runs; different seeds).

Task	Mean ± SD	95% CI
HumanPPI (Acc%)	90.66 ± 0.550	[89.98, 91.34]
Metal Ion Binding (Acc%)	77.01 ± 0.200	[76.76, 77.26]
EC ($F_{\max}$)	0.891 ± 0.005	[0.885, 0.897]
DeepLoc Subcellular (Acc%)	84.79 ± 0.080	[84.69, 84.89]

Table S5. Raw and Holm–Bonferroni–adjusted p-values for DynamicsPLM across tasks. Raw p-values are from two-tailed paired t-tests across proteins. We report the maximum Holm–Bonferroni–adjusted p across these seven comparisons for the task.

Task	Raw p-values (7 baselines)	Max Holm–Bonferroni p
HumanPPI	0.003, 0.007, 0.009, 0.010, 0.014, 0.021, 0.031	0.045
Metal Ion Binding	0.004, 0.008, 0.009, 0.011, 0.015, 0.022, 0.029	0.048
EC	0.003, 0.006, 0.008, 0.010, 0.013, 0.019, 0.027	0.040
DeepLoc (Subcellular)	0.005, 0.008, 0.009, 0.011, 0.015, 0.023, 0.033	0.048

S3.3 Reliability diagrams

As classification networks must not only be accurate but also reliable in their uncertainty estimates, calibrated confidence (Guo *et al.*, 2017) is essential for interpretability and downstream decision-making. We evaluate and plot the expected calibration error (ECE) (Guo *et al.*, 2017) for the classification tasks, using 10 equal-width bins over the confidence or predicted value range, following the calibration equation (Guo *et al.*, 2017; Levi *et al.*, 2019). DynamicsPLM consistently exhibits improved calibration over SaProt (Su *et al.*, 2024), with lower ECE in DeepLoc (0.086 vs. 0.143) and Metal Ion Binding (0.051 vs. 0.116). We observed similar trends in the additional tasks. Reliability diagrams (see Supplementary Figure S1) show that DynamicsPLM tracks the identity line more closely than SaProt across the confidence bins.

S4 Ablation tests

S4.1 Ablation: fixed vs. learned mixing weight λ

In the full DynamicsPLM, we keep the mixing weight λ fixed (shared across residues and inputs), which we found to be stable and to incur negligible overhead. As defined in Section S1.0.2:

$$\mathbf{e}_i \;=\; \lambda\,\mathbf{e}_i^{\text{dynamic}} \;+\; (1-\lambda)\,\mathbf{e}_i^{\text{seq}}$$

We ablate this choice by learning a per-residue $\lambda_i \in [0,1]$ via a lightweight gate (all other settings unchanged). The gate is implemented as `LayerNorm`(H) $\rightarrow$ `Linear`(H,H) $\rightarrow$ `GELU` $\rightarrow$ `Dropout(0.1)` $\rightarrow$ `Linear`$(H,1)$ $\rightarrow$ `Sigmoid`, where H is the embedding dimension; the final bias is initialized to -0.85 (so $\sigma(-0.85) \approx 0.30$) to conservatively favor sequence features. In all full-model experiments we set $\lambda = 0.5$.

Supplementary Table S6 shows that learning λ_i does not yield measurable gains and slightly increases run-to-run variance. Empirically, the gate converges toward a near-constant weight (low entropy across positions), indicating that a global trade-off between $\mathbf{e}^{\text{dynamic}}$ and $\mathbf{e}^{\text{seq}}$ suffices for these benchmarks; a fixed λ is therefore preferred for simplicity and stability.

Table S6. Ablation of the static–dynamic mixing weight. "Fixed λ" is the full DynamicsPLM; "Learned λ_i" uses a per-residue gate. Statistically significant results ($p < 0.05$) using a two-tailed paired t-test across test proteins are marked with an asterisk (*). The best result is in bold.

Task	Fixed λ (full)	Learned λ_i
HumanPPI (Acc%)	**90.66***	90.11
Metal Ion Binding (Acc%)	**77.01***	76.94
EC ($F_{\max}$)	**0.891***	0.887
DeepLoc Subcellular (Acc%)	**84.79***	84.24

S4.2 Ablation: mean-pooling over generated conformers

In this ablation, we replace the dynamic embedding layer with mean-pooling over the generated conformers. For each protein, we generate K conformers, similar to the dynamic embedding layer (see Section S1), and convert each to a structure-aware sequence. Then, we run the encoder once per conformer, and average the resulting embeddings across the K conformers using a mean-pooling operation.

Averaging full-sequence embeddings collapses multi-modal residue states into a single intermediate representation. Functionally distinct conformations (e.g., open vs. closed loop; ion-bound vs. unbound pocket) are blended, which can obscure the signal the classifier needs at decision time. In contrast, DynamicsPLM does not average conformers: it builds a per-residue distribution over structural tokens and feeds that distribution to the model. This retains multi-modality, closer to a local free-energy landscape, so the network can learn to emphasize the functional state when it matters instead of being forced into a single mean embedding. Even as $K \to \infty$, mean-pooling converges to an unstructured expectation over complete sentences that ignores residue-wise uncertainty the model could exploit; our distributional embedding preserves that uncertainty explicitly at each position.

Empirically (see Supplementary Table S7), mean-pooling underperforms the full DynamicsPLM on all benchmarks and shows higher run-to-run variance. This supports exposing the positional distribution directly to the encoder rather than compressing it via pre- or post-encoder averaging.

Moreover, when compared against the top-performing single-structure baseline (SaProt), mean-pooling is equal or slightly worse, indicating that indiscriminate aggregation dilutes informative states and amplifies noise from outlier/non-physiological conformers. In contrast, DynamicsPLM's selective integration of ensemble information preserves signal while maintaining lower variance, yielding consistent gains over the single-conformation baseline.

Finally, our method is computationally more efficient: it runs the encoder only once per protein, whereas mean-pooling requires K encoder passes. This yields an approximate K times reduction in wall-clock compute for the same K.

S4.3 Ablation: conformation generator

To test whether gains arise from the fusion mechanism rather than generator-specific idiosyncrasies, we compare DynamicsPLM under two conformation generators—RocketSHP (Sledzieski and Hanson, 2025) and BioEmu (Lewis *et al.*, 2025)—while holding the encoder, data splits, training protocol, and ensemble size fixed ($K = 20$).

Across benchmarks (Table S8), BioEmu closely tracks RocketSHP yet is modestly lower on all tasks, without statistically significant differences; the preserved ranking and effect sizes indicate that ensemble-conditioned fusion, not

Table S7. Ablation comparing our proposed DynamicsPLM to (i) a mean-pooling over K embeddings produced from generated conformers and (ii) a static-structure baseline (SaProt (Su *et al.*, 2024); single conformation). Statistically significant results ($p < 0.05$) via a two-tailed paired t-test across test proteins are marked with an asterisk (*). The best result is in bold.

Task	DynamicsPLM (full)	Mean-pooling	SaProt
HumanPPI (Acc%)	**90.66***	86.11	86.67
Metal Ion Binding (Acc%)	**77.01***	74.77	75.15
EC ($F_{\max}$)	**0.891***	0.879	0.876
DeepLoc Subcellular (Acc%)	**84.79***	83.44	83.19

Table S8. Ablation comparing our proposed DynamicsPLM under two conformation generators (RocketSHP (Sledzieski and Hanson, 2025), BioEmu (Lewis *et al.*, 2025)) and a static-structure baseline (SaProt (Su *et al.*, 2024); single conformation). Statistically significant results ($p < 0.05$) via a two-tailed paired t-test across test proteins are marked with an asterisk (*). The best result is in bold.

Task	DynamicsPLM (RocketSHP)	DynamicsPLM (BioEmu)	SaProt
HumanPPI (Acc%)	**90.66***	**89.44***	86.67
Metal Ion Binding (Acc%)	**77.01***	**76.54***	75.15
EC ($F_{\max}$)	**0.891***	**0.883***	0.876
DeepLoc Subcellular (Acc%)	**84.79***	**84.46***	83.19

properties of a particular sampler, drives the improvement. To mitigate concerns about leakage, we verified for BioEmu that no evaluation protein overlaps its pre-training corpus above 30% sequence identity.

These results mirror independent evaluations of SHP on ATLAS (Vander Meersche *et al.*, 2023), where RocketSHP attains lower K-divergence (van Erven and Harremoës, 2012) than BioEmu (1.089 vs. 1.923 at matched sampling budgets; lower is better) (Sledzieski and Hanson, 2025).

We further evaluate sensitivity to the number of conformations with $K \in \{5, 10, 20, 30\}$. Performance improves from $K = 5$ to $K = 10$–20, where results are most stable, while smaller values reduce conformational diversity (e.g., HumanPPI: 88.88%, EC: 0.879). Increasing K beyond this range provides no additional benefit and may introduce noisy or redundant conformations (e.g., $K = 30$: HumanPPI 88.33%, EC 0.877). These findings suggest that moderate ensemble sizes ($K = 10$–20) provide a favorable trade-off between performance and computational cost.

S4.4 Ablation: Random Perturbation Control

To distinguish meaningful conformational dynamics from noise-based regularization, we introduce a control experiment where K conformations are generated by adding zero-mean Gaussian noise to the alpha-carbon backbone coordinates of AlphaFold2 structures, with noise levels uniformly spanning 0.5–1.5 Å, following prior robustness evaluations (Cho *et al.*, 2024). These perturbed conformations are processed identically to RocketSHP-generated ensembles.

Across tasks, the random-noise baseline consistently underperforms structured conformational ensembles. On HumanPPI, accuracy drops from 90.66% to 87.22%, EC $F_{\max}$ decreases from 0.891 to 0.878, and similar trends are observed for Metal Ion Binding (77.01% vs. 75.49%) and DeepLoc (84.79% vs. 83.72%).

These results suggest that DynamicsPLM benefits from structured conformational diversity rather than arbitrary perturbations, supporting the importance of biologically meaningful dynamic ensembles.

References

Armenteros, A. (2017). Deeploc: prediction of protein subcellular localization using deep learning. *Bioinformatics*, **33**, 3387–3395.

Cho, Y., Ovchinnikov, S., and Frank, C. (2024). Enhancing protein design robustness through noise-informed sequence design. In *ICML 2024 AI for Science Workshop*.

Dallago, C., Mou, J., Johnston, K. E., Wittmann, B., Bhattacharya, N., Goldman, S., Madani, A., and Yang, K. K. (2021). FLIP: Benchmark tasks in fitness landscape inference for proteins. In *Thirty-fifth Conference on Neural Information Processing Systems Datasets and Benchmarks Track (Round 2)*.

Escobedo, N., Tunque Cahui, R. R., Caruso, G., García Ríos, E., Hirsh, L., Monzon, A. M., Parisi, G., and Palopoli, N. (2022). Codnas-q: a database of conformational diversity of the native state of proteins with quaternary structure. *Bioinformatics*, **38**(21), 4959–4961.

Escobedo, N., Monzon, A. M., Fornasari, M. S., Palopoli, N., and Parisi, G. D. (2023). Combining protein conformational diversity and phylogenetic information using codnas and codnas-q. *Current Protocols*, **3**.

Gligorijević, V., Renfrew, P. D., Kosciólek, T., Leman, J. K., Berenberg, D., Vatanen, T., Chandler, C., Taylor, B. C., Fisk, I., Vlamakis, H., Xavier, R. J., Knight, R., Cho, K., and Bonneau, R. (2021). Structure-based protein function prediction using graph convolutional networks. *Nature Communications*, **12**.

Guo, C., Pleiss, G., Sun, Y., and Weinberger, K. Q. (2017). On calibration of modern neural networks. In *Proceedings of the 34th International Conference on Machine Learning - Volume 70*, ICML'17, page 1321–1330. JMLR.org.

Hayes, T., Rao, R., Akin, H., Sofroniew, N. J., Oktay, D., Lin, Z., Verkuil, R., Tran, V. Q., Deaton, J., Wiggert, M., Badkundri, R., Shafkat, I., Gong, J., Derry, A., Molina, R. S., Thomas, N., Khan, Y. A., Mishra, C., Kim, C., Bartie, L. J., Nemeth, M., Hsu, P. D., Sercu, T., Candido, S., and Rives, A. (2025). Simulating 500 million years of evolution with a language model. *Science*, **387**(6736), 850–858.

Hu, M., Yuan, F., Yang, K. K., Ju, F., Su, J., Wang, H., Yang, F., and Ding, Q. (2022). Exploring evolution-aware & -free protein language models as protein function predictors. In *Neural Information Processing Systems*.

Humphreys, I. R., Pei, J., Baek, M., Krishnakumar, A., Anishchenko, I., Ovchinnikov, S., Zhang, J., Ness, T. J., Banjade, S., Bagde, S. R., Stancheva, V. G., Li, X.-H., Liu, K., Zheng, Z., Barrero, D. J., Roy, U., Kuper, J., Fernández, I. S., Szakal, B., Branzei, D., Rizo, J., Kisker, C., Greene, E. C., Biggins, S., Keeney, S., Miller, E. A., Fromme, J. C., Hendrickson, T. L., Cong, Q., and Baker, D. (2021). Computed structures of core eukaryotic protein complexes. *Science*, **374**(6573), eabm4805.

Levi, D., Gispan, L., Giladi, N., and Fetaya, E. (2019). Evaluating and calibrating uncertainty prediction in regression tasks. *Sensors (Basel, Switzerland)*, **22**.

Lewis, S., Hempel, T., Jiménez-Luna, J., Gastegger, M., Xie, Y., Foong, A. Y. K., Satorras, V. G., Abdin, O., Veeling, B. S., Zaporozhets, I., Chen, Y., Yang, S., Foster, A. E., Schneuing, A., Nigam, J., Barbero, F., Stimper, V., Campbell, A., Yim, J., Lienen, M., Shi, Y., Zheng, S., Schulz, H., Munir, U., Sordillo, R., Tomioka, R., Clementi, C., and Noé, F. (2025). Scalable emulation of protein equilibrium ensembles with generative deep learning. *Science*, **389**(6761), eadv9817.

Lin, Z., Akin, H., Rao, R., Hie, B., Zhu, Z., Lu, W., Smetanin, N., Verkuil, R., Kabeli, O., Shmueli, Y., dos Santos Costa, A., Fazel-Zarandi, M., Sercu, T., Candido, S., and Rives, A. (2023). Evolutionary-scale prediction of atomic-level protein structure with a language model. *Science*, **379**(6637), 1123–1130.

Monzon, A. M., Zea, D. J., Fornasari, M. S., Saldaño, T. E., Fernandez-Alberti, S., Tosatto, S. C. E., and Parisi, G. (2017). Conformational diversity analysis reveals three functional mechanisms in proteins. *PLOS Computational Biology*, **13**(2), 1–18.

Rives, A., Goyal, S., Meier, J., Guo, D., Ott, M., Zitnick, C. L., Ma, J., and Fergus, R. (2019). Biological structure and function emerge from scaling unsupervised learning to 250 million protein sequences. *Proceedings of the National Academy of Sciences of the United States of America*, **118**.

Sledzieski, S. and Hanson, S. (2025). Rocketshp: Ultra-fast proteome-scale prediction of protein dynamics. *bioRxiv*.

Su, J., Han, C., Zhou, Y., Shan, J., Zhou, X., and Yuan, F. (2024). Saprot: Protein language modeling with structure-aware vocabulary. In *The Twelfth International Conference on Learning Representations*.

van Erven, T. and Harremoës, P. (2012). Rényi divergence and kullback-leibler divergence. *IEEE Transactions on Information Theory*, **60**, 3797–3820.

van Kempen, M., Kim, S., Tumescheit, C., Mirdita, M., Lee, J., Gilchrist, C. L., Söding, J., and Steinegger, M. (2022). Fast and accurate protein structure search with foldseek. *Nature Biotechnology*, **42**, 243 – 246.

Vander Meersche, Y., Cretin, G., Gheeraert, A., Gelly, J.-C., and Galochkina, T. (2023). Atlas: protein flexibility description from atomistic molecular dynamics simulations. *Nucleic Acids Research*, **52**(D1), D384–D392.

Xu, M., Zhang, Z., Lu, J., Zhu, Z., Zhang, Y., Ma, C., Liu, R., and Tang, J. (2022). Peer: A comprehensive and multi-task benchmark for protein sequence understanding. In *Thirty-sixth Conference on Neural Information Processing Systems Datasets and Benchmarks Track*.

Yang, K. K., Zanichelli, N., and Yeh, H. (2022). Masked inverse folding with sequence transfer for protein representation learning. *Protein Engineering, Design and Selection*, **36**, gzad015.

Zhang, Z., Xu, M., Jamasb, A. R., Chenthamarakshan, V., Lozano, A., Das, P., and Tang, J. (2023a). Protein representation learning by geometric structure pretraining. In *The Eleventh International Conference on Learning Representations*.

Zhang, Z., Wang, C., Xu, M., Chenthamarakshan, V., Lozano, A. C., Das, P., and Tang, J. (2023b). A systematic study of joint representation learning on protein sequences and structures.
